# The injection of maternal B complex vitamin during the transition period: The impact on performance, thyroid hormones levels and immunological parameters in the Sannen goats and their kids, as well as the faeces status of newborn kids

**DOI:** 10.1002/vms3.1561

**Published:** 2024-07-30

**Authors:** Mohammad Asadi, Maryam Hatami, Homa Mohammadi Fard

**Affiliations:** ^1^ Department of Animal and Poultry Nutrition Animal Science Faculty Gorgan University of Agricultural Sciences and Natural Resources Gorgan Iran; ^2^ Department of Animal Science Faculty of Agriculture University of Tabriz Tabriz Iran; ^3^ Faculty of Veterinary Medicine Islamic Azad University, Babol Branch Babol Iran

**Keywords:** B complex vitamin, immunological parameters, newborn kid, Sannen goat, thyroid hormones, transition period

## Abstract

**Background:**

It is proven that B vitamins through promote a wide range of metabolic pathways in animals as cofactors improve animal performance.

**Objectives:**

This study was conducted to investigate the impacts of maternal B complex vitamin injection on performance and plasma parameters in goats and their offspring, as well as the faeces status of newborn kids.

**Methods:**

In this research, the pregnant goats (3 years old) were randomly divided into two groups: the control group (without B complex vitamin injection) and the B complex vitamin group (5 mL B complex vitamin injection per animal). The animals were injected with 5 mL B complex vitamin twice during the transition period (5 weeks pre‐ and 5 weeks post‐kidding). The goats during the transition period and kids on days 10, 20 and 30 were weighed. Feed intake by goats and consumption of milk and starter in kids were recorded daily. The dry matter digestibility by kids was tested by collecting samples of faeces and feed for 5 days in the last week. Chemical analysis was determined using the AOAC method. The kids’ faeces were prepared daily during the study. The blood samples of goats and newborn kids were taken 7 days after kidding. Then, levels of B group vitamin, as well as concentrations of liver enzymes, thyroid hormones and immunological parameters, were determined in plasma of goat and their offspring. In addition, concentrations of glucose and insulin were measured in goat plasma (Asadi et al., 2024).

**Results:**

According to results, the performances of goats and their offspring, as well as kids’ faeces status, were improved by maternal B complex vitamin injection (*p* < 0.0001). The levels of cobalamin, pyridoxine, thiamine, folic acid, nicotinic, pantothenic and unconjugated pteridine increased in the plasma of goats and their kids in the B complex vitamin group compared with the control group during the transition period (*p* < 0.0001). Injection of maternal B complex vitamin raised the plasma levels of triiodothyronine and tetraiodothyronine, immunoglobulin G and immunoglobulin M in goats and their offspring (*p *< 0.0001). Higher levels of glucose and lower levels of insulin were determined in the goats injected with B complex vitamin (*p* < 0.0001).

**Conclusions:**

These results suggest that maternal B complex vitamin injection is required for the improvement of performance, health status and the blood plasma parameters in pregnant goats and their kids.

## Graphical abstract

The maternal B complex vitamin injection led to improving performance and blood plasma metabolites of goat and newborn kids. Moreover, faecal consistency score, number of kids with diarrhoea and average days of diarrhoea were improved.

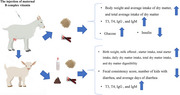

## 1 INTRODUCTION

Environmental parameters, including maternal nutrients and hormones, as well as inherited genetic profiles, can significantly influence foetal development during pregnancy. These factors may have a long‐lasting impact on the health and growth of the offspring throughout life (Geraghty et al., 2015). An increase in inflammation, oxidative stress, adipose tissue mobilization and metabolic disorders (such as ketosis, fatty liver and milk fever) are all associated with the perinatal period, that is the last 3 weeks prepartum through the first 3 weeks post‐partum (Coleman et al., 2021). Further, the rapid growth of the uterus and fetus during pregnancy, coupled with lactation, results in a rise in nutrient intake during the transition period (NRC, 2012). Research shows that B vitamin helps maintain normal appetites (Ongan & Yuksel, 2017) and animal performance (Ren et al., 2022). In addition to their role as cofactors in thyroid hormone synthesis (Habeeb & Gad, 2019), they also play a significant role in immunity (Hosomi & Kunisawa, 2017; Suzuki & Kunisawa, 2015).

According to recent studies, injection of maternal B complex vitamin during the transition period improved performance, metabolic diseases, as well as haematological and antioxidant parameters in goats and their offspring (Asadi et al., 2023). Injection of vitamin B12 improved growth rate and increased body dimensions of goat. This effect was more pronounced on the length and height than on bone width development. Thus, it is essential to keep serum vitamin B12 levels above 350 pg/mL in young growing goats to ensure reasonable growth rates (Kadim et al., 2006).

In the B complex vitamin group, eight water‐soluble vitamins are known: thiamin (vitamin B1), riboflavin (vitamin B2), niacin (vitamin B3), pantothenic acid (vitamin B5), pyridoxine (vitamin B6), biotin (vitamin B8), folic acid (vitamin B9) and cobalamin (vitamin B12). It is known that B vitamins are widely distributed in feeds, and their effects can be felt throughout the body. By functioning as coenzymes, they provide energy to the body (Bellows et al., 2012).

Therefore, the objective of this study was to examine the effects of maternal vitamin B complex injection on performance, liver enzymes, thyroid hormones and immune of the Sannen goat and their offspring, as well as kids’ faeces consistency during the transitional period.

## 2 MATERIALS AND METHODS

### 2.1 Animal management and experimental treatments

The study was conducted in the research station, in Gorgan, Iran in April of 2023. This study used 40 pregnant Sannen goats (3 years, 48 ± 2.7 kg). The goats were synchronized by a CIDER, then they mated with males. The goats were sonographed before treatment. The goats were kept in individual boxes of 1 × 1 m^2^ with a concrete floor covered with chaff. The animals were fed the same diet during the study (5 weeks before kidding and 5 weeks after kidding). Diet was formulated according to NRC (2012), which is reported in Table 1. Water was provided ad libitum in the buckets in each box individually. Experimental treatments were as follows: T_1_ (control): without vitamin B complex injection; T_2_: vitamin B complex injection. In this study, 5 mL of B complex vitamin (B Co‐ject Injection Rooyan, each millilitre of the solution contains: thiamine: 10 mg; riboflavin: 4 mg; pyridoxine: 4 mg; cyanocobalamin: 10 mg; niacin amide: 50 mg; dexpanthenol: 5 mg) were injected intramuscularly twice in each animal during the transition period (5 weeks pre‐ kidding and 5 weeks post‐kidding), according to the manufacturer's recommendation. The goats gave birth during 48 h, and 40 kids were born. Povidone iodine was used to disinfect the kids’ navels after parturition, and which were kept in individual pens. During the early hour after kidding, 10% of the body weight of pooled herd colostrum was fed by nipple bottle (Asadi et al., 2023).

**TABLE 1:** Diet components of goat.

Ingredient (%) DM basis‐pre‐partum		Ingredient (%) DM basis‐post‐partum	
Wheat straw	5.7	Corn silage	34
Alfalfa	32	Alfalfa	30
Corn silage	30	Corn grain	19.75
Corn grain	18.5	Soybean meal	7.75
Soybean meal	7.2	Beet pulp sugar	2
Beet pulp sugar	1	Wheat bran	2.7
Wheat bran	2.9	Fat powder	2.8
Fat powder	1.5	Calcium carbonate	0.42
Calcium carbonate	0.7	Salt	0.33
Salt	0.3	Mineral‐vitamin supplement	0.25
Mineral‐vitamin supplement	0.2		

Chemical composition		Chemical composition	
Nutrients diet	Amount	Nutrients diet	Amount
Metabolizable energy (Kcal/kg)	2.44	Metabolizable energy (Kcal/kg)	2.54
Crude protein (%)	14.40	Crude protein (%)	14.40
Crude fat (%)	4.10	Crude fat (%)	5.20
Non‐fibrous carbohydrates (%)	32.80	Non‐fibrous carbohydrates (%)	32.10
NDF (%)	44.20	NDF (%)	40.90
Starch (%)	21.60	Starch (%)	25.00
Ash (%)	7.88	Ash (%)	8.40
Calcium (%)	1.42	Calcium (%)	0.89
Phosphorus (%)	0.71	Phosphorus (%)	0.52

### 2.2 Performance and faeces consistency

The goats were weighed 5 weeks before kidding, during kidding, and 5 weeks after kidding. Moreover, kids on days 10, 20 and 30 were weighed. Feed intake by goats, as well as consumption of milk and starter of kids, were recorded daily. The total dry matter intakes form milk and starter by kids. For 5 days, final week of study, samples of faeces and feed were collected to determine digestibility of dry matter by kids, and chemical analysis was determined using the AOAC (2000) method. The kids’ faeces were evaluated daily during the study. Stool scores were examined based on 1: hard and consistent; 2: soft and loose; 3: loose and watery; 4: watery with some blood and 5: watery with blood and mucus (Khan et al., 2007).

### 2.3 Plasma blood parameters

In this study, 7 days after kidding, blood samples were taken from the jugular vein of goats and their offspring to measure plasma biochemical parameters. Afterwards, blood samples were transferred into K2EDTA tubes (anticoagulant) (Sarstedt Polska). Then, plasma was prepared by centrifuging blood samples at 3000 *g* for 10 min at room temperature (Toghdory et al., 2023). After that, plasma samples were stored at −20°C until they could be analysed.

To determine activities of aspartate aminotransferase (AST) and alanine aminotransferase (ALT), 100 mL of plasma were mixed with 1000 mL of solutions R1 and R2 (Kit Cont. P.L. 97203232, Pars Azmoun Company) at 37°C. The light absorption was read after 1 min using a photometric spectrometer (UV–Vis model 365 LAMBDA, Perkinelmer) with an emission wavelength of 340 nm. So, the stopwatch is started and after 1, 2 and 3 min, the difference light absorption from the first absorption was determined. Finally, the average light difference was multiplied by 1985 (Toghdory et al., 2023).

Level of alkaline phosphatase (ALP) was assessed by Pars Azmoun Company Kit (Kit Cont. P.L. 97203232). Twenty millilitre of plasma with 1000 mL of solutions R1 and R2 were mixed at 37°C. The light absorption was recorded after 1 min with an emission wavelength of 405 nm using a photometric spectrometer (UV–Vis model 365 LAMBDA, Perkinelmer). The stop watch is started. In the times of 1, 2 and 3 min, the difference light absorption was assessed from the first absorption. Then, the average light difference was multiplied by 3433 (Toghdory et al., 2023).

The concentrations of thyroid hormones, triiodothyronine (T_3_) and tetraiodothyronine (T_4_) were assayed by the enzyme‐linked immunosorbent assay (ELISA), Pars Peyvand (Cat. No. DG. T_3_/T_4_ 0.01). In this method, 25 mL of standard solutions, control and serum samples were added to the wells. Then, 100 mL of conjugated enzyme solution was added to each well and incubated for 15 s. The plate was covered by a special label and incubated at room temperature for 60 min. Next, the wells were washed four times with 300 mL of washing solution. Dye solution of 100 mL was added to each well and incubated at room temperature for 20 min. Then, 100 mL of stopping solution was added to each well and incubated for 15 s. Finally, the light absorption at an emission wavelength of 450 nm by ELISA (ELX808. TEX‐Bio, BioTek Instruments) (Asadi et al., 2024).

We measured concentration of insulin‐like growth factor‐I (IGF‐I) in plasma using immunoassay kits from Diagnostic Systems Laboratories, Inc. (Yu et al., 1999). The method for IGF‐I was immunoradiometric assay, which employed in each kit two specific anti‐bodies to form the sandwich complex. The ranges of calibrators were 4.5–640 ng/mL for IGF‐I. For IGF‐I, the specimens were pre‐treated (1:1000) with an acid‐ethanol extraction buffer to separate IGFs from their binding proteins.

The level of tumour necrosis factor (TNF‐α) was assayed by the Pars Azmon Company kit (Kit Cont. 96RYNS). First, 50 mL of the standards and 50 mL of the samples were added to the wells. After 2 h incubation on a shaker, the plates were washed three times using washing solution. Five millilitre of conjugated antibody was added to all wells and incubated on a shaker for 1 h at room temperature. Again, the plates were washed three times. Five millilitre of Avidin‐HRP solution was added to all the wells and incubated for 30 min at room temperature on a shaker. Then, using the washing solution, the plates were washed five times. Five millilitre of the substrate was added to all the wells and incubated for 15 min at room temperature on a shaker. Finally, 5 mL of stopping solution was added to all the wells and the light absorption for the plasma samples was measured by ELISA (ELX808. TEX‐Bio, BioTek Instruments) at an emission wavelength of 455 nm (Asadi et al., 2024).

To assess the levels of immunoglobulin G (IgG) and immunoglobulin M (IgM) were used the Pars Azmon Company kit. First, 10 mL of sample were added to 500 mL of solution R1. After 3 min incubation at 37°C, light absorption was recorded by ELISA (ELX808. TEX‐Bio, BioTek Instruments) at an emission wavelength of 340 nm. Then, 50 mL of solution R2 was added to all the wells and incubated for 10 min at 37°C. The second light absorption was recorded (Asadi et al., 2024).

Creatine phosphokinase (CPK) concentration was analysed via Pars Azmoun Company Kit (Kit Cont. P.L. 97203232). In this regard, 20 mL of plasma were blend with 1000 mL of solutions R1 and R2 at 37°C. After 1 min, the light absorption was noted by a photometric spectrometer (UV–Vis model 365 LAMBDA, Perkinelmer) with an emission wavelength of 340 nm. Next, the stopwatch is started and after 1, 2 and 3 min, the difference of light absorption was assessed from the first absorption. Then, the average light difference was multiplied by 4127 (Asadi et al., 2024).

The evaluation of ceruloplasmin (CP) was based on its oxidase activity using o‐dianisidine dihydrochloride (ODD) as a substrate, as described by Schosinsky et al. (1974). Briefly, serum samples were incubated in duplicate at 37°C in 7.88 mM ODD in 0.1 M acetate buffer, pH 5.0. The light absorption variation in relation to the colour intensity of the sample was measured using a spectrophotometer (UV–Vis model 365 LAMBDA, Perkinelmer) against a blank (see above) at 540 nm after 5 and 15 min of incubation using 9 M H_2_SO_4_ to stop the enzymatic reaction. The CP oxidase activity was expressed in units per litre (U/L) in terms of consumed substrate and calculated as the difference between the two absorbance values.

To determine creatinine concentration, 50 mL of plasma with 1000 mL of solutions R1 and R2 (Pars Azmoun Company Kit) at 37°C were blended. After 60 s incubation, the first light was absorption, and after 120 s, the second light absorption (UV–Vis model 365 LAMBDA, Perkinelmer) with an emission wavelength of 500 nm was recorded (Toghdory et al., 2023).

Pars Peyvand Company Kit was used to assess lactate dehydrogenase (LDH) level. For this aim, 20 mL of sample were added to 800 mL of solution R1 and incubated for 5 min at 37°C. Then, 200 mL of solution R2 was added and mixed. After 1 min, the light absorption (UV–Vis model 365 LAMBDA, Perkinelmer) with an emission wavelength of 340 nm was read. Immediately, the stopwatch was started and after 1, 2 and 3 min the light absorption was measured (Toghdory et al., 2023).

Pars Azmoun Company Kit (Kit Cont. P.L: 64780179) was used to assess glucose concentration. Ten millilitre of plasma was mixed with 1000 mL of solution R1. After 20 min incubation at room temperature, the light absorption (UV–Vis model 365 LAMBDA, Perkinelmer) with an emission wavelength of 546 nm was recorded (Asadi et al., 2024).

Commercial kit from Monobind Inc. was used to measure insulin concentration. Briefly, 25 mL of blood plasma were added to wells. Next, 50 mL of insulin enzyme reagent were added and incubated for 60 min at room temperature. After that, the plate was washed twice with washing buffer, and after adding the solution containing the two mentioned substrates and incubation for 15 min, 50 mL of terminating solution were added. The light absorption of the samples was read at an emission wavelength of 450 nm using a photometric spectrometer (UV–Vis model 365 LAMBDA, Perkinelmer). The insulin concentration of the samples was calculated according to the insulin standard curve (Asadi et al., 2024).

### 2.4 Statistical analysis

Levene's and Kolmogorov–Smirnov and Shapiro–Wilk tests were used to examine the data for homogeneity of variances and normality of data distributions, respectively. Then, data were analysed using the GLM procedure of SAS (version 9.1, SAS Institute Inc., SAS Campus Drive) based on a completely randomized design of two treatments and 20 replicates. Duncan's multiple range test was used to compare means at the probability 5% level.

The following statistical model was used to analyze the data:

$$Y_{\mathrm{ijk}}=\mu +A_{i}+E_{\mathrm{aik}}+B_{j}+AB_{\mathrm{ij}}+E_{\mathrm{bijk}}$$

where Y_ijk_ is the observed *k* variable in the *i*th treatment and *j*th time. μ is the overall mean of the observed variables. A_i_ is the effect of *i*th treatment. E_aik_ is the main error. B_j_ is the effect of *j*th time. AB_ij_ is the effect of interaction between *i*th treatment and *j*th time. E_bijk_ is the random residual error.

## 3 RESULTS

### 3.1 Performance of goats

Table 2 shows the performance of goats during the study. According to the results, a higher body weight rate was shown in the B complex vitamin group than in the control group at 5 weeks prior kidding, during kidding and 5 weeks post‐kidding, *p* value as recorded in Table 2 (*p* < 0.0001). In addition, the average intake of dry matter during 5 weeks prior‐ and 5 post‐kidding, as well as the total average intake of dry matter in the B complex vitamin group, demonstrated a significant increase compared to the control group (*p* < 0.0001).

**TABLE 2:** The effect of maternal B complex vitamin injection on goat performance.

Performance	Control	B complex	SEM	*p*‐value
Birthweight of kids (kg)	3.12^b^	3.55^a^	0.269	0.0001
Pre‐partum weight of goats (kg)	53.52^b^	55.57^a^	2.333	0.0005
Goat weight at kidding (kg)	47.18^b^	49.61^a^	2.004	0.0270
Goat weight after kidding (kg)	45.57^b^	48.38^a^	1.986	0.0001
Pre‐partum DM intake (g/h)	2001.79^b^	2149.88^a^	59.982	0.0001
Post‐partum DM intake (g/h)	1793.36^b^	1968.76^a^	42.774	0.0001
Total DM intake (g)	1897.57^b^	2059.32^a^	60.203	0.0001

*Note*: Different letters indicate significant differences (*p* < 0.05) between groups.

### 3.2 Performance of kids

Statistical analysis of kid's performance parameters is reported in Table 3. In this regard, maternal injection of B complex vitamin led to a remarkably increase in birthweight and body weight on days 10, 20 and 30, and total body weight in kids (*p* < 0.0001). However, trial treatments unaffected daily gain and total daily gain in kids during the study (*p* > 0.05). On the other hand, feeding performance, including milk offered, daily and total starter intakes, daily and total dry matter intakes and dry matter digestibility, was significantly increased by the injection of maternal B complex vitamin in kids (*p* < 0.0001). Nevertheless, the feed conversion ratio between the groups had no significant difference (*p* > 0.05).

**TABLE 3:** The effect of maternal B complex vitamin injection on kid performance.

Performance	Control	B complex	SEM	*p*‐value
Daily gain (1–10 days, g/day)	199.00	208.00	9.748	0.5611
Body weight (10 day, kg)	5.11^b^	5.63^a^	0.218	0.0012
Daily gain (11–20 days, g/day)	181.00	192.00	12.655	0.4870
Body weight (20 day, kg)	6.92^b^	7.55^a^	0.487	0.0001
Daily gain (21–30 days, g/day)	198.00	209.00	17.696	0.5129
Body weight (30 day, kg)	8.90^b^	9.64^a^	0.794	0.0001
Total daily gain (g/day)	192.67	203.00	10.296	0.748
Total body weight (kg)	5.78^b^	6.09^a^	0.622	0.0142
Milk offered (1–30 days, g/day)	524.12^b^	601.29^a^	32.114	0.0001
Daily starter intake (g/day)	201.78^b^	224.88^a^	17.884	0.0005
Total starter intake (kg)	6.05^b^	6.74^a^	0.121	0.0001
Daily dry matter intake (g/day)	237.36^b^	266.39^a^	19.874	0.0061
Total dry matter intake (kg)	7.12^b^	7.99^a^	0.2482	0.0001
Feed conversion ratio	1.23	1.31	0.044	0.6212
Dry matter digestibility (%)	59.16^b^	67.88^a^	3.829	0.0014

*Note*: Different letters indicate significant differences (*p* < 0.05) between groups.

### 3.3 Faces consistency

Results of kids’ faeces consistency are presented in Table 4. Maternal B complex vitamin injection led to an improvement in faecal consistency score and decreased the number of kids with diarrhoea and average days of diarrhoea in kids (*p* < 0.0001).

**TABLE 4:** The effect of maternal B complex vitamin injection on kid faeces consistency.

Faeces consistency	Control	B complex	SEM	*p*‐value
Faecal consistency score	1.68^a^	1.20^b^	0.109	0.0084
Number of kids with diarrhoea	4.75^a^	2.00^b^	0.225	0.0001
Average days of diarrhoea	3.00^a^	1.75^b^	0.167	0.0001

*Note*: Different letters indicate significant differences (*p* < 0.05) between groups.

### 3.4 B vitamins concentration

Table 5 shows the results related to the concentrations of B vitamin groups in blood plasma. According to data, the levels of B vitamins, including cobalamin, pyridoxine, thiamine, folic acid, nicotinic, pantothenic and unconjugated pteridine, increased in the plasma of goats and their offspring by maternal B complex injection as compared to the control group during the transition period (*p* < 0.0001).

**TABLE 5:** The effect of maternal B complex vitamin injection on concentrations of B vitamin group in blood plasma of goats and their offspring.

B vitamins (μg/mL)	Control	B complex	SEM	*p*‐value
**Goats**				
Cobalamin	0.24^b^	0.55^a^	0.012	0.0001
Pyridoxine	27.04^b^	44.00^a^	2.006	0.0001
Thiamine	6.18^b^	11.61^a^	0.814	0.0001
Folic acid	8.21^b^	16.05^a^	1.001	0.0001
Nicotinic	61.07^b^	79.07^a^	4.991	0.0001
Pantothenic	506.92^b^	1011.77^a^	52.866	0.0001
Unconjugated pteridine	14.26^b^	40.95^a^	2.018	0.0001
**Kids**				
Cobalamin	0.17^b^	0.43^a^	0.010	0.0001
Pyridoxine	23.77^b^	35.92^a^	3.096	0.0125
Thiamine	4.61^b^	8.28^a^	0.901	0.0001
Folic acid	7.04^b^	12.85^a^	1.202	0.0001
Nicotinic	56.11^b^	74.96^a^	4.556	0.0001
Pantothenic	449.77^b^	881.61^a^	42.869	0.0001
Unconjugated pteridine	11.72^b^	29.84^a^	1.882	0.0001

*Note*: Different letters in each row indicate a significant difference (*p* < 0.05) between treatments.

### 3.5 Liver enzymes and thyroid hormones

In Table 6, the results related to liver enzymes and plasma thyroid hormones are reported. Maternal injection of B complex vitamin increased concentrations of T_3_ and T_4_ in blood plasma of goats and their offspring compared to the control group (*p* < 0.0001). However, activities of AST, ALT, ALP and T_3_/T_4_ were not affected by trial treatments in the blood plasma of goats and their offspring (*p* > 0.05).

**TABLE 6:** The effect of maternal B complex vitamin injection on plasma levels of liver enzymes and thyroid hormones.

Liver enzymes and thyroid hormones	Control	B complex	SEM	*p*‐value
**Goats**				
Aspartate aminotransferase (U/L)	56.61	54.80	4.339	0.5582
Alanine aminotransferase (U/L)	15.97	16.04	1.166	0.3131
Alkaline phosphatase (U/L)	222.09	229.75	24.556	0.5802
Triiodothyronine (nmol/L)	8.97^b^	9.62^a^	1.011	0.0001
Tetraiodothyronine (nmol/L)	101.97^b^	120.61^a^	19.052	0.0001
Triiodothyronine/Tetraiodothyronine	11.36	12.53	0.848	0.1211
**Kids**	^N^			
Aspartate aminotransferase (U/L)	62.77	59.91	5.002	0.6268
Alanine aminotransferase (U/L)	16.90	16.14	0.976	0.4229
Alkaline phosphatase (U/L)	219.15	225.65	30.114	0.2289
Triiodothyronine (nmol/L)	7.65^b^	8.60^a^	0.977	0.0001
Tetraiodothyronine (nmol/L)	98.55^b^	108.60^a^	14.241	0.0001
Triiodothyronine/Tetraiodothyronine	12.88	12.62	1.084	0.2440

*Note*: Different letters indicate significant differences (*p* < 0.05) between groups.

### 3.6 Immunological parameters

Table 7 shows the immunological parameters of goats and their newborn kids. Statistical analysis revealed that concentrations of IgG and IgM were increased in the blood plasma of goats and their offspring by maternal B complex vitamin injection compared to the control group (*p* < 0.0001). Nonetheless, other immunological parameters, including IGF‐1, TNF‐α, CPK, CP, creatinine and LDH, did not show significant differences between experimental groups in goats and their newborn kids (*p* > 0.05).

**TABLE 7:** The effect of maternal B complex vitamin injection on plasma immunological parameters.

Immunological parameters	Control	B complex	SEM	*p*‐value
**Goats**				
Insulin‐like growth factor (ng/mL)	51.66	52.61	2.911	0.6940
Tumour necrosis factor (pg/mL)	114.11	110.92	8.554	0.6262
Immunoglobulin G (mg/mL)	12.06^b^	14.61^a^	1.021	0.0001
Immunoglobulin M (mg/mL)	0.66^b^	0.81^a^	0.033	0.0001
Creatine phosphokinase (μ/L)	58.69	60.01	1.771	0.7441
Ceruloplasmin (mg/dL)	21.14	20.81	2.891	0.6988
Creatinine (mg/dL)	0.66	0.67	0.011	0.8484
Lactate dehydrogenase (μ/L)	247.67	250.61	14.551	0.4718
**Kids**				
Insulin‐like growth factor (ng/mL)	49.51	48.69	2.561	0.6004
Tumour necrosis factor (pg/mL)	109.68	107.55	9.004	0.6582
Immunoglobulin G (mg/mL)	11.81^b^	12.99^a^	0.898	0.0001
Immunoglobulin M (mg/mL)	0.64^b^	0.79^a^	0.101	0.0001
Creatine phosphokinase (μ/L)	55.64	57.61	2.001	0.4114
Ceruloplasmin (mg/dL)	19.60	18.91	1.961	0.4855
Creatinine (mg/dL)	0.69	0.71	0.089	0.4898
Lactate dehydrogenase (μ/L)	241.55	248.01	16.721	0.5579

*Note*: Different letters indicate significant differences (*p* < 0.05) between groups.

### 3.7 Levels of glucose and insulin

**FIGURE 1:**
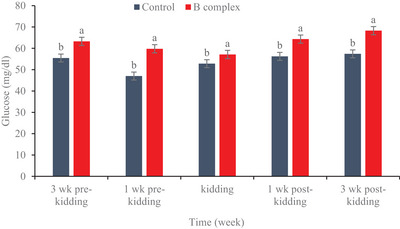
The effect of maternal B complex vitamin injection on plasma glucose level in goat.

**FIGURE 2:**
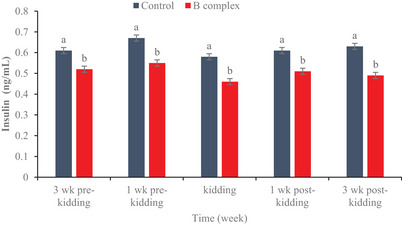
The effect of maternal B complex vitamin injection on plasma insulin concentration in goat.

Concentrations of glucose and insulin are shown in Figures 1 and 2, respectively. Higher (*p* < 0.0001) level of glucose and lower (*p* < 0.0001) level of insulin were found in the B complex vitamin group compared to the control group were observed.

## 4 DISCUSSION

Ruminants require B vitamin group for essentially all metabolic processes (Spears & Weiss, 2014). As B vitamins are enzymatic cofactors or metabolic constituents, they are essential to metabolic processes such as Kreb's cycle, gluconeogenesis, as well as metabolism of carbohydrate, fatty and protein (Al‐Abbasy, 2013; McDowell, 2000; Zimmerly & Weiss, 2001). In this study, by increasing appetite (Ongan & Yuksel, 2017) and reducing oxidative stress (Dalto & Matte, 2017) in the transition period, B vitamin injection led to a reduction in energy‐negative balance by improving animal feed intake. Accordingly, the increase in feed intake and nutrient digestibility in the B complex vitamin group was responsible for improving body weight in goats and their offspring. It is also likely that an increase in kid's body weight was related to positive effects of B complex vitamin on energy utilization efficiency (Ren et al., 2022). In agreement with this study, lambs fed niacin‐supplemented diets consumed more feed and grew faster (Hashemian et al., 2020). Feed fortification of B‐vitamins (riboflavin, pantothenic acid, niacin, B12 and folic acid) improved the pig performance (Coelho, 2001). In contrast, injection and supplemental folic acid did not increase animal body weight, weight gain and total body weight gain (Karim & Omar, 2021).

The B vitamin group, especially folates and vitamin B12, is essential for nucleotide synthesis and cell growth, affecting the immune system, and reducing diarrhoea (Taneja et al., 2013). Currently, research regarding the effect of B vitamin group on ruminant's diarrhoea status is scarce, and more research is necessary.

By entering into the enzyme system, B vitamin group favours the thyroid's vesicular epithelium's ability to utilize thyroid stimulating hormone )TSH( (Elghamry & Badawi, 1965). The increase in T_3_ and T_4_ concentrations in goats and their kids that received B complex vitamin may be due to that B complex vitamin stimulates the production of TSH, which helps the thyroid gland to produce thyroid hormones (Habeeb & Gad, 2019). A higher levels of thyroid hormones were observed in goats receiving biotin supplements (Habeeb & Gad, 2019), similar to the current results.

It has been reported that B vitamin group plays a vital role in maintaining immune homeostasis, cofactors and coenzymes in various metabolic pathways (Hosomi & Kunisawa, 2017; Suzuki & Kunisawa, 2015). Thiamine is a cofactor for several enzymes, including pyruvate dehydrogenase and α‐ketoglutarate dehydrogenase, both are involved in the tricarboxylic acid (TCA) cycle (Frank, 2007). Furthermore, in the TCA cycle and in fatty acid oxidation (also called oxidative metabolism), riboflavin and its active forms (flavin adenine dinucleotide and flavin mononucleotide) act as cofactors (Huskisson et al., 2007). Shortage of riboflavin, biotin and pantothenic acid defeats the activity of acyl‐CoA dehydrogenases involved in the oxidation of fatty acids to generate acetyl‐CoA, which is used by mitochondria to produce ATP via the TCA cycle. Fatty acid oxidization by generation of acetyl‐CoA and its entry into TCA cycle is involved in the activation, differentiation and proliferation of immune cells (Almeida et al., 2016). As a result, riboflavin appears to be involved in regulating the oxidation of fatty acids, which affects the differentiation and function of immune cells. In the present study, maternal B complex vitamin injection increased blood plasma levels of IgM and IgG in goats and their offspring through the mechanisms mentioned. Similar, the humoral immune response, measured by IgG antibody titres at days 14 and 28 post‐infection, was improved via B vitamin injection in calves recuperating from feed deprivation (Dubeski et al., 1996). Short‐term supplementation of pyridoxine recovered serum antibody (IgG and IgM) production in pyridoxine deficient mice (Kumar & Axelrod, 1968).

Possible mechanisms involved in the increase of plasma glucose concentration in goats injected with B complex vitamin can be as follows: Biotin is a cofactor for the enzymes propionyl‐CoA carboxylase and pyruvate carboxylase (McDowell, 2000), which are involved in glucose synthesis and increase glucose production (Zimmerly & Weiss, 2001). Furthermore, this increase may relate to the role of niacin in increasing energy use and blood sugar levels (Al‐Abbasy, 2013). Maintaining normal blood glucose levels is possible with pyridoxine. To maintain normal blood sugar levels when caloric intake is low, pyridoxine converts stored carbohydrates into glucose (Albert et al., 2008; Herrmann et al., 2007).

## 5 CONCLUSION

The results demonstrate that B complex vitamin could be used as a maternal injection for improving performance, thyroid hormone secretion and immune status of goats and their offspring, as well as promoting the health of newborn kids. Consequently, in this study, it is recommended to use 5 mL of injectable B complex vitamin in pregnant goats.

## AUTHOR CONTRIBUTIONS

**Homa Mohammadi Fard and Kamel Amozadeh Araee**: Conceptualization; methodology; investigation; project administration; funding acquisition; review. **Mohammad Asadi**: Conceptualization; methodology; investigation; formal analysis; review and editing. **Maryam Hatami**: Conceptualization; methodology; investigation; writing–original draft; writing–review and editing.

## CONFLICT OF INTEREST STATEMENT

The authors declare no conflicts of interest.

### ETHICS STATEMENT

All experimental procedures involving animals were approved by the Animal Welfare and Ethics Committee of Gorgan University Agricultural Science and Natural re‐sources, Gorgan, Iran.

### PEER REVIEW

The peer review history for this article is available at https://publons.com/publon/10.1002/vms3.1561.

## Data Availability

The data supporting the findings of this study are available on request from the corresponding author.
